# Effects of stocking density on the growth performance, mitophagy, endocytosis and metabolism of *Cherax quadricarinatus* in integrated rice–crayfish farming systems

**DOI:** 10.3389/fphys.2022.1040712

**Published:** 2022-11-28

**Authors:** Yin Dong, Rui Jia, Yiran Hou, Weixu Diao, Bing Li, Jian Zhu

**Affiliations:** ^1^ Wuxi Fisheries College, Nanjing Agricultural University, Wuxi, China; ^2^ Key Laboratory of Integrated Rice-Fish Farming Ecology, Ministry of Agriculture and Rural Affairs, Freshwater Fisheries Research Center, Chinese Academy of Fishery Sciences, Wuxi, China

**Keywords:** *Cherax quadricarinatus*, stocking density, transcriptome analysis, growth performance, oxidative stress, metabolism

## Abstract

Red claw crayfish (*Cherax quadricarinatus*) is an economic freshwater shrimp with great commercial potential. However, the suitable stocking density of *C. quadricarinatus* is still unclear in integrated rice–crayfish farming system. Thus, this study aimed to investigate the effects of stocking density on growth performance, mitophagy, endocytosis and metabolism of *C. quadricarinatus.* The *C. quadricarinatus* was reared at low density (LD, 35.73 g/m^2^), middle density (MD, 71.46 g/m^2^) and high density (HD, 107.19 g/m^2^) in an integrated rice–crayfish farming system. After 90 days of farming, the growth performance of *C. quadricarinatus* significantly decreased in the MD and HD groups relative to that in the LD group. The HD treatment caused oxidative stress and lipid peroxidation at the end of the experiment in hepatopancreas. Transcriptome analysis showed that there were 1,531 DEGs (differently expressed genes) between the LD group and HD group, including 1,028 upregulated genes and 503 downregulated genes. KEGG (Kyoto Encyclopedia of Genes and Genomes) enrichment analysis indicated that the DEGs were significantly enriched in endocytosis and mitophagy pathways. Meanwhile, four lipid metabolism pathways, including biosynthesis of unsaturated fatty acids, fatty acid biosynthesis, glycerolipid metabolism and glycerophospholipid metabolism, exhibited an upregulated tendency in the HD group. In conclusion, our data showed that when the stocking density reached up to 207.15 g/m^2^ in HD group, the growth performance of *C. quadricarinatus* was significantly inhibited in this system. Meanwhile, the data indicated that *C. quadricarinatus* may respond to the stressful condition *via* activating antioxidant defense system, endocytosis, mitophagy and metabolism-related pathways in hepatopancreas.

## Introduction

In aquaculture practice, semi-intensive and intensive aquaculture modes are commonly selected by farmers to obtain higher economic benefits, where the farmed animals maintain a high stocking density (Roy et al., 2022). As one of the critical factors, stocking density influences the water quality, growth, survival, behavior, and health of farmed aquatic animals (Ani et al., 2022; Hossain et al., 2022). Studies on stocking density have been conducted in many aquatic species, such as turbot (*Scophthalmus maximus*) (Liu B et al., 2019), rainbow trout (*Oncorhynchus mykiss*) (Zahedi et al., 2019), Pacific white shrimp (*Litopenaeus vannamei*) (Deng et al., 2019) and Siberian sturgeon (*Acipenser baerii*) (Aidos et al., 2018). A variety of adverse effects are caused in aquatic animals when they are stocked in a high density. It has been reported that the growth performance and survival rate of aquatic animals are decreased under a high stocking density due to the intensification of interspecific competition and/or the deterioration of water quality (Trenzado et al., 2006; Yuan et al., 2018). High stocking density, as a stressor, may disturb the balance of homeostasis in farmed aquatic animals, which further negatively impacts on immune function and physiological status, even increases the susceptibility to diseases (Lin et al., 2015; Ellison et al., 2018; Liu B et al., 2019; Ellison et al., 2020). An increasing stocking density induces oxidative stress leading to high catalase (CAT) activity and malonaldehyde (MDA) content in channel catfish (*Ictalurus punctatus*) (Refaey et al., 2022). In addition, some studies demonstrated that high stocking density could decrease digestive enzyme activity (Liu et al., 2018) and change body composition (Aidos et al., 2018). Hence, an appropriate stocking density is vital in aquaculture, which may take into consideration both welfare of animal and economic benefits of farming.

Transcriptomic analysis is considered as a reliable tool and has been widely used to assess the molecular mechanism of abnormal physiological change caused by adverse stimulus in aquatic animals. Under high stocking density, transcriptomic analysis reveals adverse alterations in muscle quality and immune function of grass carp (*Ctenopharyngodon idellus*) (Zhao et al., 2019). According to transcriptomic analysis, Ellison et al. (2018) demonstrated that rearing density significantly impacts susceptibility of Nile tilapia (*Oreochromis niloticus*) to the oomycete *Saprolegnia parasitica*. In large yellow croaker (*Larimichthys crocea*), transcriptomic analysis showed that short crowding stress can induce an immune response, but long-term high stocking density may suppress the immunity (Sun et al., 2017). It should be noted that transcriptomic analysis can provide more molecular function information, which contributes to understanding the mechanism of rearing density in aquatic animals.

In China, there are multiple aquaculture models including pond farming, lake/reservoir farming, indoor recirculating aquaculture and integrated rice-aquatic animals farming. Among these, integrated rice-aquatic animals farming has expanded rapidly in the last 10 years, and the farming area is 2.56 million hectares and aquatic production reaches up to 3.25 million tons in 2020 (National Fesheries Technology Extension Center, 2021). It has been considered as a sustainable strategy that improves the utilization of land and water resources, provides food for human, and alleviates environmental pollution resulted from agricultural production (Bashir et al., 2020). In the rice-aquatic animals co-culture system, the excrement of aquatics animals as fertilizer can be utilized by rice to meet the growth requirement for nitrogen and phosphorus, while weeds, insects and plankton can be eaten by aquatic animals as food, which reduces the input of commercial diet (Vromant et al., 2001; Tsuruta et al., 2010). It has been reported that rice-aquatic animals co-culture system has higher ecosystem service value compared with rice monoculture (Liu et al., 2020). In farming practice, various aquatic animals, such as common carp (*Cyprinus carpio*) (Liu et al., 2022), Chinese mitten crab (*Eriocheir sinensis*) (Jiang et al., 2021), red swamp crayfish (*Procambarus clarkia*) (Si et al., 2018) and Chinese soft-shelled turtle (*Trionyx sinensis*) (Wu et al., 2021), have been co-cultured with rice. It is worth noting that existing research majorly focuses on the assessment of ecological and economic values, microbial diversity and soil nutrition in the integrated rice-aquatic animals farming system (Si et al., 2018; Liu et al., 2020), but the suitable stocking density of aquatic animals and the effects of stoking density on physiological function are rarely evaluated.

*C. quadricarinatus*, also known as red claw crayfish, has considerable potential for commercial culture due to high growth rate and well tolerance to stressful conditions (Naranjo-Páramo et al., 2004). There are two farming models for *C. quadricarinatus* including pond monoculture and rice-crayfish co-culture in China. The suitable stocking density of *C. quadricarinatus* in the pond monoculture has been reported, and the effect of high stocking density on growth performance was evaluated (Pinto and Rouse, 1996; Naranjo-Páramo et al., 2021). However, in rice-crayfish co-culture system, the suitable stocking density has not been reported. In addition, it is unclear that how high density affects the physiological, biochemical and molecular variations of *C. quadricarinatus*. Therefore, in this study, we set different rearing densities in the integrated rice-crayfish system, and then compared the growth performance and biochemical parameters among different stocking densities after 90 days farming. Further, we evaluated the underlying mechanism of stress induced by high stocking density in *C. quadricarinatus via* transcriptomic analysis.

## Materials and methods

### Animals, experimental design and sampling

*C. quadricarinatus* used in the study were purchased from Zhejiang Freshwater Fisheries Research Institute (Huzhou, China). The experiment was carried out at the farm of Freshwater Fisheries Research Center (Jingjiang, China). The integrated rice-crayfish farming system consists of a rice field (360 m^2^) and a canal refuge (0.8 m in depth, 40 m^2^) (Supplementary Figure S1). In the system, rice seeding (Nangeng 5,055) was transplanted in the middle of June 2021, and the rice was harvested in early November. The management of rice fields was based on local agricultural practice experience. Basic fertilizer (16% nitrogen, 8% phosphorus and 16% potassium) was applied before rice transplantation, and no fertilizer was used after *C. quadricarinatus* farming.

In the rice-crayfish system, we reared three densities of *C. quadricarinatus*: low density (LD, 35.73 g/m^2^), middle density (MD, 71.46 g/m^2^) and high density (HD, 107.19 g/m^2^). Each density included three repetitions. The average initial weight was 14.29 ± 1.05 g/crayfish. The experiment lasted 90 days from 22 July 2021. During the experiment, the crayfish were fed on a commercial feed (crude protein ≥30%, crude fat ≥3%, crude fiber ≤8%, crude ash ≤18%, total phosphorus ≥1%, and calcium 1%–3.5%) once every day. The daily feed ration was adjusted according to crayfish weight (1%–2% of weight). During the trial period, the water quality maintains within a reasonable range, such as 0.19–0.48 mg/L total ammonia nitrogen, 0.01–0.04 mg/L nitrite, 23.4–32.1°C temperature and 3.21–5.08 mg/L dissolved oxygen. The amount of fed diet and mortality of crayfish were recorded.

To assess the growth performance, the body weight was measured every 30 days *via* randomly catching 20% of the crayfish in each group. After 90 days of farming, 45 individuals in each group were sampled at random for biochemical and transcriptome analyses. In biochemical analysis, the hepatopancreas of five individuals was pooled into a sample, while 15 individuals’ hepatopancreas were mixed into a sample to sequence transcriptome. All samples were stored in liquid nitrogen temporarily, and then stored for a long time under −80°C. The use of crayfish in this study was approved by the Freshwater Fisheries Research Centre (FFRC), Wuxi, China. All experiment operations were performed according to the requirement of animal welfare.

### Growth performance

The growth performance was assessed by calculating specific growth rate (SGR, % day^−1^), weight gain ratio (WGR, %) and survival rate (SR, %) under three different densities. The calculation formulas are presented as follows:

$$SGR=100\times \left({lnW}_{2}-{lnW}_{1}\right)/n$$

$$WGR=100\times \left(W_{2}-W_{1}\right)/W_{1}$$

SR=100×final number /initial number

Where, W_1_ and W_2_ are initial weight (g) and final weight (g), n is the days of the feeding trial.

### Determination of oxidative stress parameters

Oxidative stress parameters including total antioxidant capacity (T-AOC), malondialdehyde (MDA), superoxide dismutase (SOD), catalase (CAT), glutathione (GSH) and glutathione peroxidase (GPx) were determined in hepatopancreas. The determination of all parameters was conducted according to the method described by the manufacturers. In addition, total protein (TP) was detected to calculate the levels of oxidative stress parameters. The commercial kits for TP, T-AOC, SOD and GSH were provided by Nanjing Jiancheng Bioengineering Institute (Nanjing, China). The kits of CAT and MDA were purchased by Beyotime Biotechnology (Shanghai, China). The kit of GPx was ordered from Grace Biotechnology Co., Ltd (Suzhou, China).

### RNA extraction, cDNA library construction, and transcriptome sequencing

The hepatopancreas from the LD and HD groups was used to transcriptome analysis. Total RNA of hepatopancreas was isolated using TRIzol reagent (Invitrogen, Carlsbad, CA, United States). RNA concentration and integrality was measured using Qubit2.0 Fluorometer (Life Technologies, CA, United States) and Agilent 2,100 Bioanalyzer, respectively (Agilent Technologies, CA, United States). The RNA was used to synthesize cDNA and then construct DNA libraries by PCR amplification according to a standard procedure. The libraries were sequenced using Illumina Novaseq6000 (Gene *Denovo* Biotechnology Co., Guangzhou, China). The raw data of transcriptome sequences have been submitted to NCBI Sequence Read Archive (SRA) database (NO. PRJNA884003).

To ensure data quality, low-quality reads in the raw data were filtered by fastp (version 0.18.0) (Chen et al., 2018). The value of Q20 (the base quality score ≥20), Q30 (the base quality score ≥30), and GC (GC content in clean reads) of clean reads were counted. The filtered reads were assembled by Trinity (version 2.8.4) (Grabherr et al., 2011), and the integrity of assemble was evaluated by Benchmarking universal Single-Copy Orthologs (BUSCO). Assembled unigenes were annotated *via* nr (Non-Redundant Protein Sequence Database), SwissProt, KEGG (Kyoto Encyclopedia of Genes and Genomes) and COG/KOG (Clusters of Orthologous Groups of proteins) databases. The unigene expression was calculated and normalized to RPKM (Reads Per Kilobase of transcript per Million mapped reads) by RSEM (Li and Dewey, 2011). Principal component analysis (PCA) was used to evaluate the relationship of samples.

The differently expressed genes (DEGs) was selected using DESeq2 (version 1.20.0) (Love et al., 2014) with the threshold value: false discovery rate (FDR) < 0.05 and fold change (FC) ≥2. All DEGs were annotated to GO (Gene Ontology) and KEGG databases to conduct enrichment analysis. Furthermore, gene set enrichment analysis (GSEA) was used to assess the differences of genes in important KEGG pathways (Subramanian et al., 2005).

### Validation of Differently expressed genes by quantitative real-time PCR

To verify the reliability of RNA-seq, the key genes related to endocytosis and mitophagy in hepatopancreas were selected and detected *via* quantitative real-time PCR (qPCR). Total RNA of hepatopancreas from the LD and HD groups was isolated using TRIzol reagent (Takara Biomedical Technology Co., Ltd, Beijing, China) according to the manufacturer’s instructions. The isolated RNA was used to synthesize cDNA using a commercial kit (Takara, RR047A). The TB Green Premix EX Taq ™ kit (Takara, RR820A) was used to amplify sequence of target gene. The *β*-actin was selected as a reference gene, and the relative expression of target gene was calculated by the 2^−ΔΔCq^ method (Livak and Schmittgen, 2001). The specific primers are shown in Supplementary Table S1.

### Statistical analysis

SPSS (version 25.0) software was used to conduct statistical analysis in this study. All values were presented as mean ± SE (standard error). The data of growth performance and antioxidant parameters were analyzed by one-way ANOVA with LSD post-hoc test, and the relative levels of mRNA between the LD group and the HD group were analyzed by *t*-test. The normal distribution and homogeneity of the variances was assessed by Shapiro-Wilk test and Levene test, respectively. Pearson test was used for correlation analysis between the qPCR data and RNA-seq data. Differences were considered to be significant if the *p*-value < 0.05.

## Results

### Growth performance parameters

**FIGURE 1:**
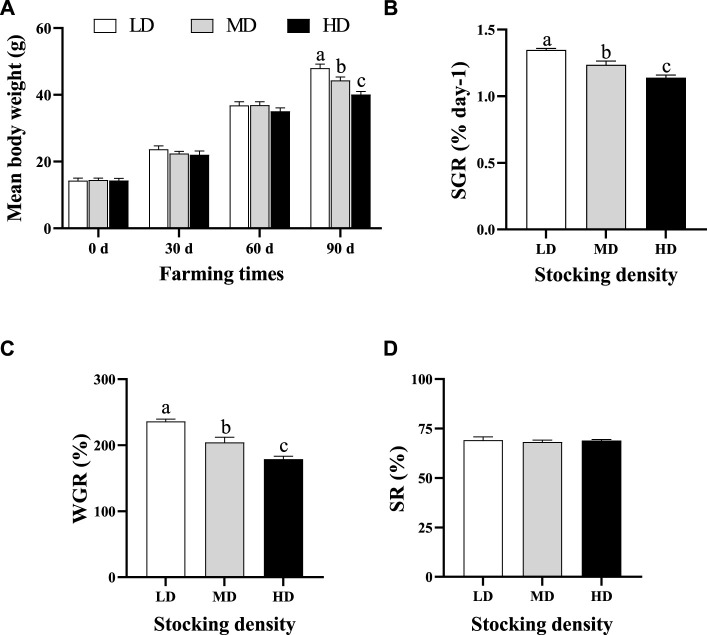
Mean body weight **(A)**, specific growth rate **(B)**, weight gain ratio **(C)** and survival rate **(D)** of *C. quadricarinatus* under different densities in an integrated rice–crayfish farming system. Values are presented as means ± SE. Means with different superscripts denote significant differences (*p* < 0.05). LD, low stocking density; MD, middle stocking density; HD, high stocking density.

The variation of body weight, SR, SGR and WGR of *C. quadricarinatus* are presented in Figure 1. The average body weight of *C. quadricarinatus* exhibited an upward trend in different groups as the farming time increased. After 90 days of farming, the average body weight was lower in the MD and HD groups than that in the LD group (*p* < 0.05). Meanwhile, the SGR and WGR significantly decreased in the MD and HD groups relative to that in the LD group (*p* < 0.05). However, the SR showed similar change among different groups (*p* > 0.05).

### Antioxidant parameters

**FIGURE 2:**
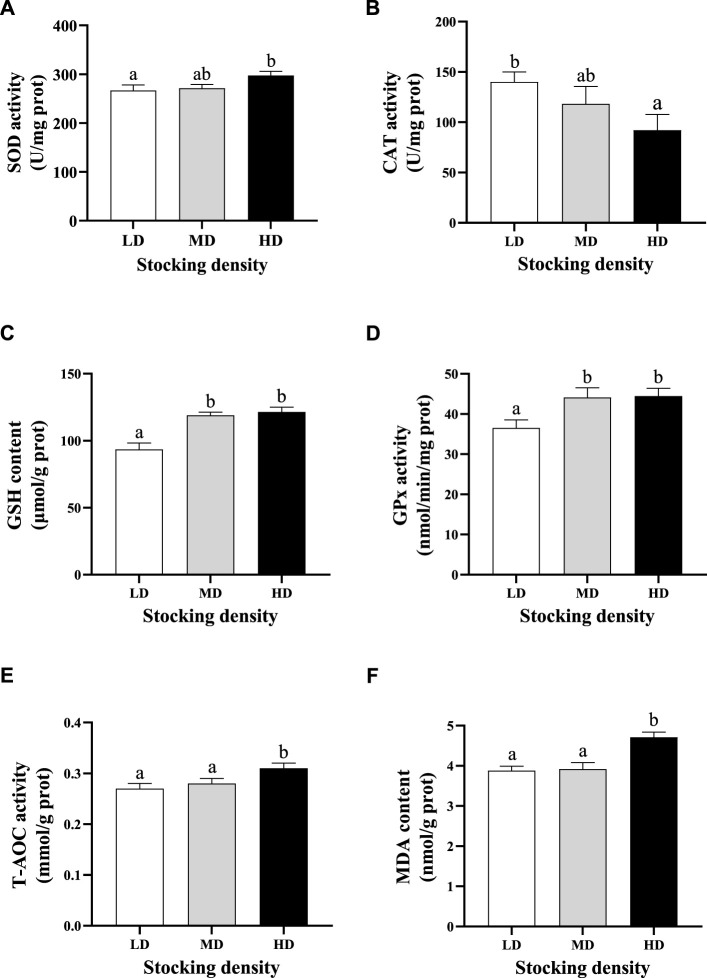
Antioxidative parameters in hepatopancreas of *C. quadricarinatus* under different densities in an integrated rice–shrimp farming system after 90 days. Values are presented as means ± SE (*n* = 9). **(A)** SOD; **(B)** CAT; **(C)** GSH; **(D)** GPx, **(E)** T-AOC; **(F)** MDA. Means with different superscripts denote significant differences (*p* < 0.05). LD, low stocking density; MD, middle stocking density; HD, high stocking density.

At the end of the trial, significant differences in antioxidant ability of *C. quadricarinatus* were observed among different stocking densities (Figure 2). The activities of SOD, CAT and GPx and the contents of MDA and GSH were higher in the HD group than those in the LD group (*p* < 0.05) after 90 days of farming, whereas the CAT activity was lower in the HD group than that in the LD group (*p* < 0.05). In addition, the levels of GSH and Gpx were also significantly increased in the MD group compared with the LD group (*p* < 0.05; Figures 2C,D).

### Transcriptome sequencing and analysis of differently expressed genes

In order to better understand the adverse effects of high stocking density, we analyzed the genes expression profile in hepatopancreas *via* RNA-seq. After filtering of raw reads, 41,402,944 (99.64%)—47,175,044 (99.66%) clean reads were obtained, which were assembled into unigenes for further analysis. The results of base quality score are as follow: the values of Q20, Q30, and GC were 97.75%–98.05%, 93.58%–94.37% and 44.71%–48.25%, respectively. The mapped ratio was greater than 92.54% (Table 1).

**TABLE 1:** Valid data used in transcriptome analysis.

Samples	Raw reads	Clean reads	Q20 (%)	Q30 (%)	GC (%)	Total maped (%)
LD-1	46,379,574	46,172,628 (99.55%)	97.91	94.08	48.08	92.96
LD-2	42,423,984	42,184,242 (99.43%)	97.83	93.91	48.25	92.80
LD-3	44,891,590	44,647,100 (99.46%)	98.05	94.37	47.70	93.35
HD-1	47,334,822	47,175,044 (99.66%)	97.97	94.08	44.73	92.82
HD-2	41,551,364	41,402,944 (99.64%)	97.75	93.58	44.71	92.54
HD-3	44,255,976	44,104,972 (99.66%)	97.94	94.03	44.79	92.91

Q20 and Q30, the base quality score (Q score) was no less than 20 and 30, respectively, in clean reads. GC, GC, content in clean reads; LD, low density group; HD, high density group.

**FIGURE 3:**
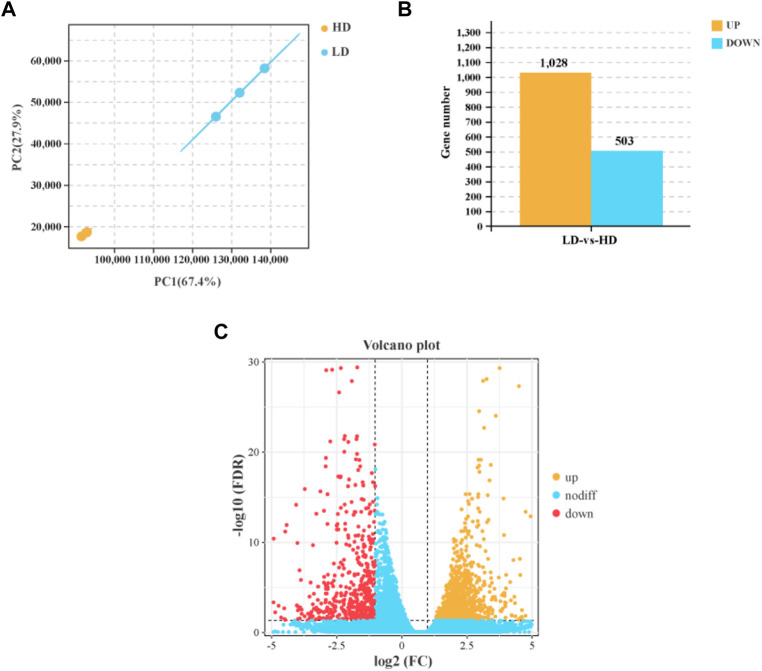
Differently expressed genes (DEGs) in the hepatopancreas of *C. quadricarinatus* between the LD and HD group. **(A)** The correlation of samples between the LD and HD groups. **(B)** The number of DEGs in the hepatopancreas between the LD and HD groups. **(C)** Volcano plot of DEGs between the LD and HD groups.

The result of PCA showed that the LD group and HD group were distinctly separated, and samples had higher correlation within group (Figure 3A). In hepatopancreas, a total of 1,531 DEGs were identified between the LD group and HD group, including 1,028 upregulated genes and 503 downregulated genes (Figures 3B,C).

### GO enrichment analysis of differently expressed genes

**FIGURE 4:**
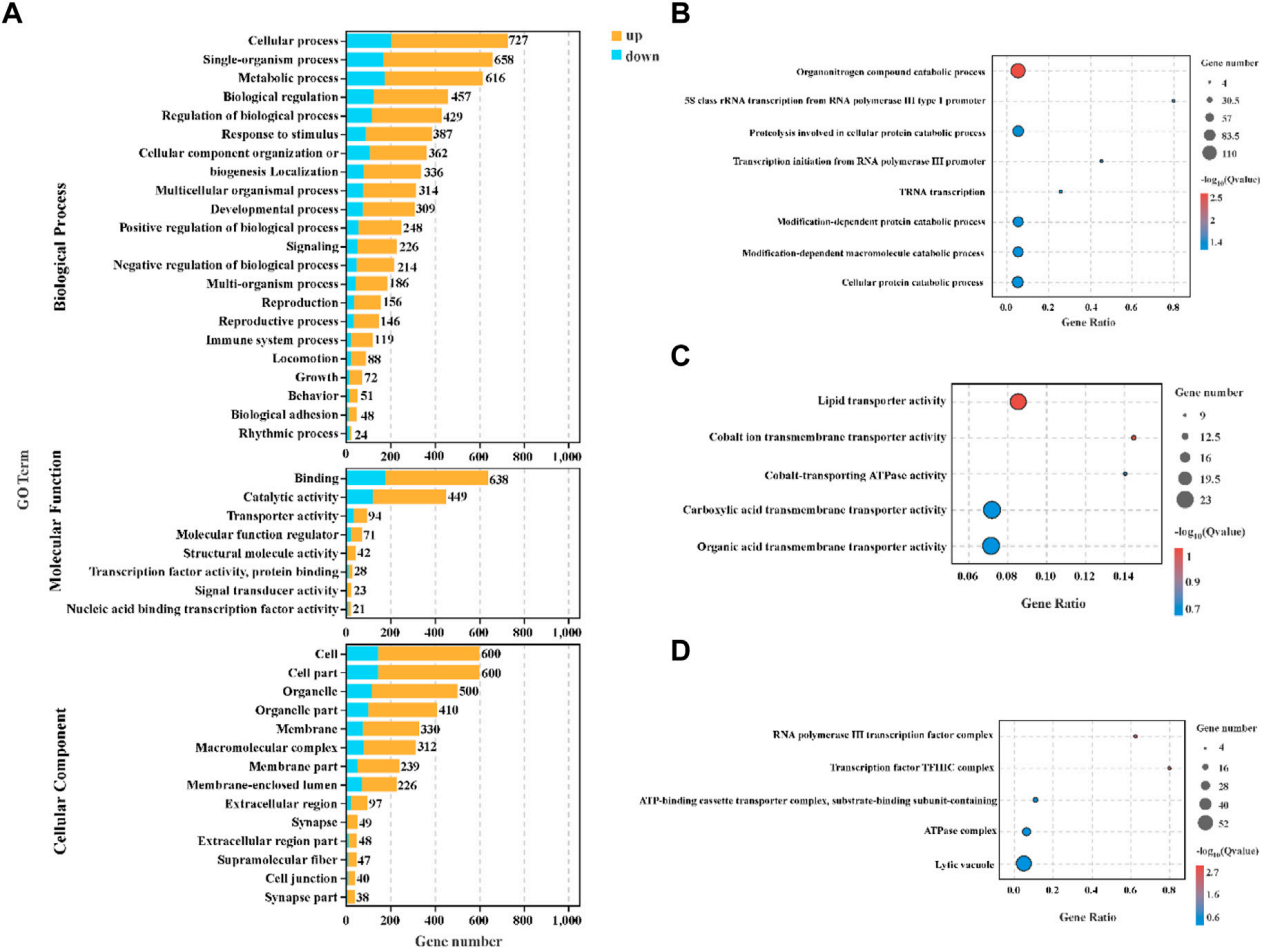
GO enrichment analysis in the hepatopancreas of *C. quadricarinatus* under two stocking densities (LD-vs-HD). **(A)** GO enrichment terms of DEGs in three ontologies. **(B)** The top 8 GO terms in the biological process. **(C)** Top 5 GO terms in the molecular function. **(D)** The top five GO terms in the cellular components.

All DEGs were subjected to GO enrichment analysis to identify significantly enriched biological process, molecular function and cellular component (Figure 4). In the biological process category, the DEGs were enriched mainly in organonitrogen compound catabolic process (*p*.adjust = 0.003), proteolysis involved in cellular protein catabolic process (*p*.adjust = 0.032), modification-dependent protein catabolic process (*p*.adjust = 0.040), modification-dependent macromolecule catabolic process (*p*.adjust = 0.040) and cellular protein catabolic process (*p*.adjust = 0.040) (Figure 4B). In the molecular function category, binding and catalytic activity involved the most DEGs (Figure 4A), and lipid transporter activity (*p*.adjust = 0.090) and cobalt ion transmembrane transporter activity (*p*.adjust = 0.090) were the top two GO terms (Figure 4C). In the cellular component category, the DEGs mainly participated in cell, cell part and organelle (Figure 4A), and RNA polymerase III transcription factor complex (*p*.adjust = 0.002) and transcription factor TFIIIC complex (*p*.adjust = 0.003) were the top two GO terms (Figure 4D).

### KEGG enrichment analysis of differently expressed genes

**FIGURE 5:**
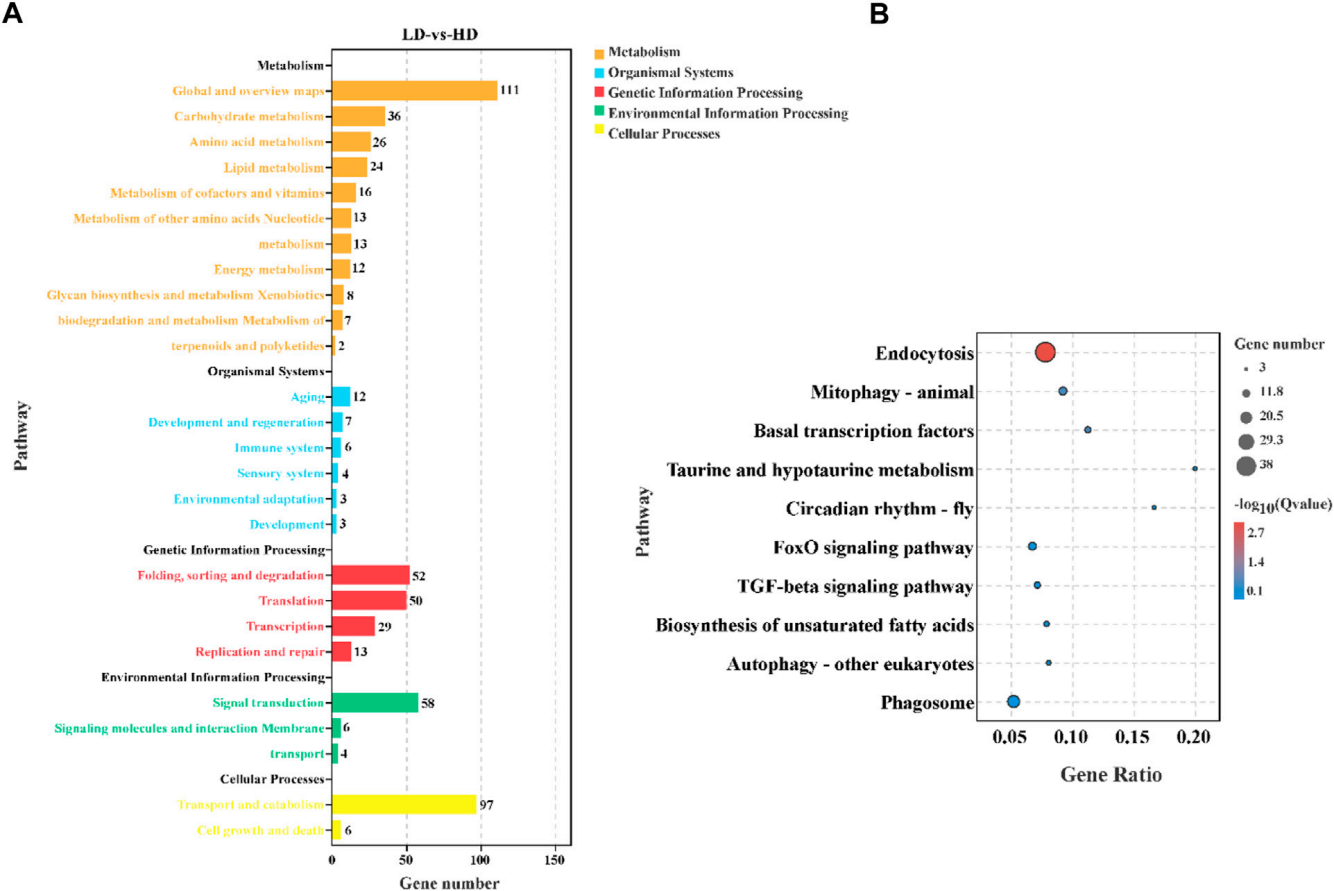
KEGG enrichment analysis in the hepatopancreas of *C. quadricarinatus* under two stocking densities (LD-vs-HD). **(A)** The result of DEGs enrichment in the KEGG. **(B)** The top 10 enriched KEGG pathways.

To understand biological function and key signaling pathways, the DEGs were enriched in KEGG database (Figure 5). The result indicated that annotated DEGs were mapped to 124 specific KEGG pathways, and metabolism was the most abundant KEGG A class (Figure 5A). The top 10 enriched KEGG pathways were presented in Figure 5B. It was worth nothing that the DEGs principally enriched in immune function (e.g., endocytosis, mitophagy-animal, autophagy-other eukaryotes and phagosome) and metabolism function (e.g., taurine and hypotaurine metabolism and biosynthesis of unsaturated fatty acids). In addition, the FoxO signaling pathway and TGF-bata signaling pathway were also affected by different stocking densities.

### Change of mitophagy pathway

**FIGURE 6:**
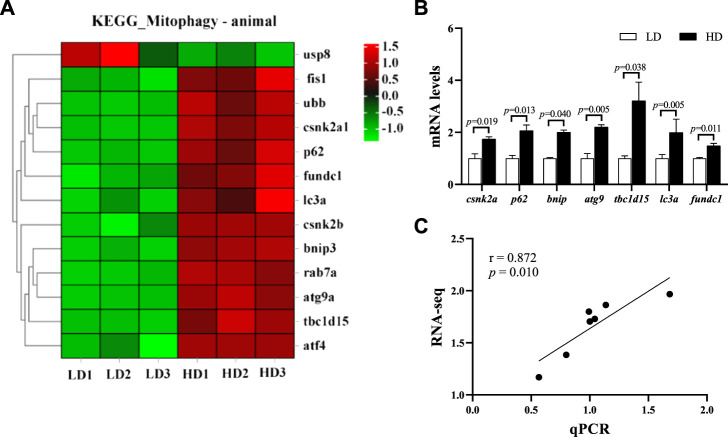
Changes of mitophagy pathway in the hepatopancreas of *C. quadricarinatus* between the LD group and the HD group. **(A)** DEGs enriched in mitophagy pathway. **(B)** Mitophagy-related gene expression measured by qPCR, values are presented as means ± SE (*n* = 3) **(C)** The correlation between the qPCR data and RNA-seq data.

At the end of the trial, 13 genes in mitophagy pathway were significantly changed, including 12 upregulated genes and 1 downregulated gene (*p* < 0.05; Figure 6A). The qPCR data showed that 7 key genes regulated mitophagy pathway, including casein kinase II subunit alpha (*csnk2a*), sequestosome-1 (*p62*), Bcl-2 nineteen kilodalton interacting protein 3 (*bnip3*), autophagy-related gene 9A (*atg9*), TBC1 domain family member 15-like isoform X2 (*tbc1d15*), microtubule-associated proteins 1A/1B light chain 3A (*lc3a*) and FUN14 domain-containing protein 1 (*fundc1*) were also significantly upregulated in the HD group (*p* < 0.05; Figure 6B), which was significantly consistent with the RNA-seq data (r = 0.872, *p* = 0.010; Figure 6C).

### Change of endocytosis pathway

**FIGURE 7:**
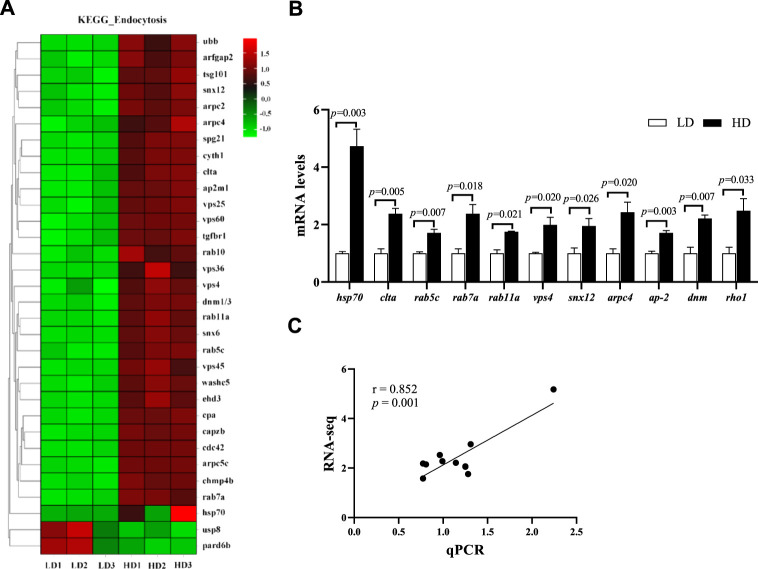
Changes of endocytosis pathway in the hepatopancreas of *C. quadricarinatus* between the LD group and the HD group. **(A)** DEGs enriched in endocytosis pathway. **(B)** Endocytosis-related gene expression measured by qPCR, values are presented as means ± SE (*n* = 3). **(C)** The correlation between the qPCR data and RNA-seq data.

After 90 days of farming, the endocytosis pathway in the hepatopancreas of *C. quadricarinatus* was significantly changed. KEGG enrichment analysis showed that a total of 32 DEGs were enriched in the endocytosis pathway, including 30 upregulated genes and 2 downregulated genes (Figure 7A). Further, the mRNA levels of 11 key genes involved in the endocytosis pathway, including heat shock protein 70 (*hsp70*), clathrin light chain (*clta*), ras-related protein Rab-5C (*rab5c*), *rab7a*, *rab11a*, suppressor protein of bem1/bed5 double mutants (*vps4*), sorting nexin-12 (*snx12*), actin-related protein 2/3 complex subunit 4 (*arpc4*), adaptor protein 2 complex subunit mu (*ap-2*), dynamin superfamily protein (*dnm*) and ras-like GTP-binding protein (*roh1*), were also upregulated in the HD group compared with the LD group (*p* < 0.05; Figure 7B). Meanwhile, correlation analysis indicated that the RNA-seq data were significantly consistent with the qPCR data (r = 0.852, *p* = 0.001; Figure 7C).

### Change of lipid metabolism pathways

**FIGURE 8:**
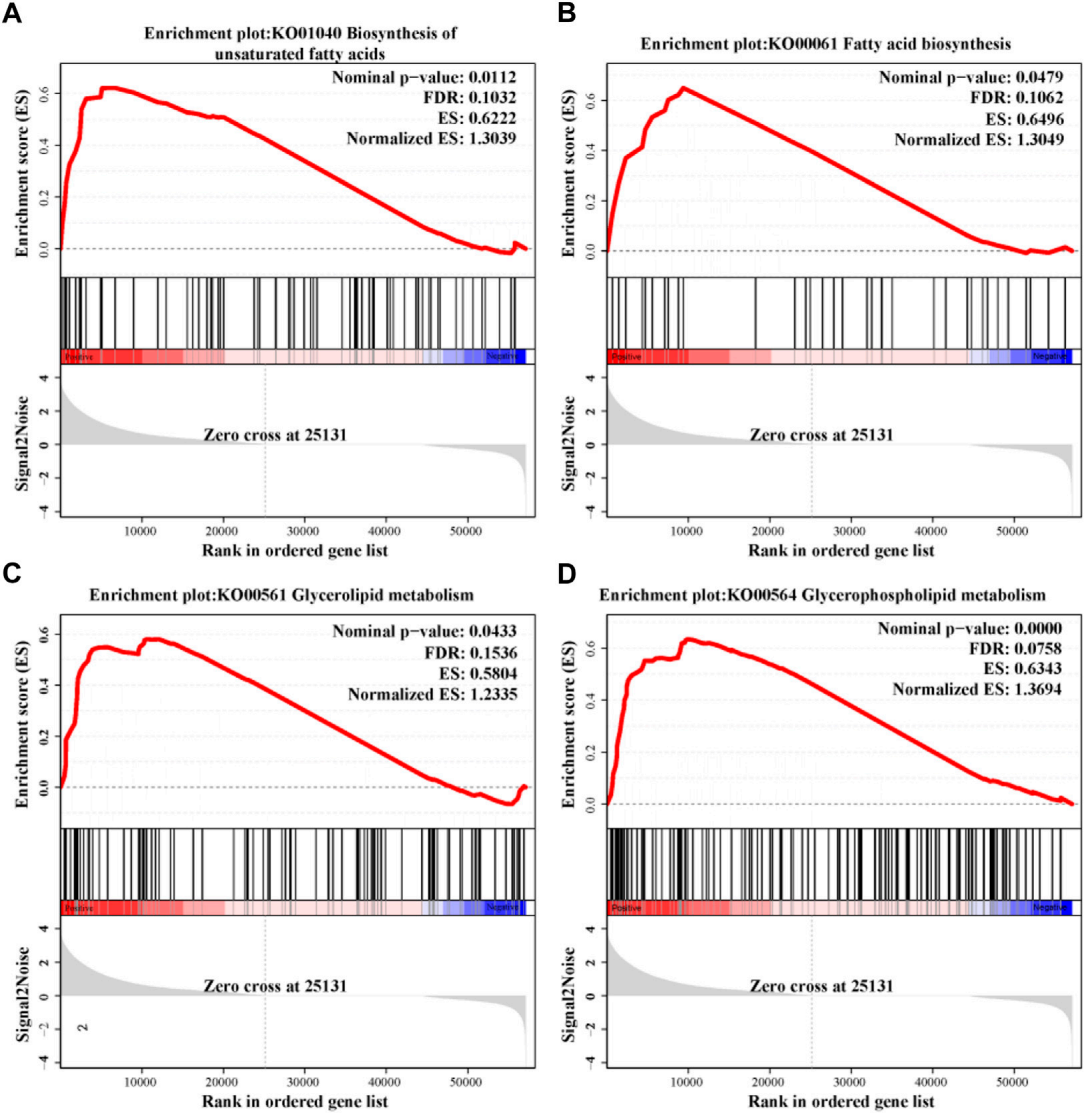
Changes in lipid metabolism pathways identified using GSEA in the hepatopancreas of *C. quadricarinatus* under two stocking densities (LD-vs-HD). **(A)** Biosynthesis of unsaturated fatty acids. **(B)** Fatty acid biosynthesis. **(C)** Glycerolipid metabolism. **(D)** Glycerophospholipid metabolism. The |Normalized ES| > 1, nominal *p*-value < 0.05 and FDR <0.25 in each gene set were set as threshold values for statistical significance.

The result of GSEA indicated that the lipid metabolism function was significantly influenced by stocking density (Figure 8). Under high stocking density, four lipid metabolism-related pathways, including biosynthesis of unsaturated fatty acids (KO01040), fatty acid biosynthesis (KO00061), glycerolipid metabolism (KO00561) and glycerophospholipid metabolism (KO00564) were more likely to be upregulated.

## Discussion

### Effect of stocking density on growth performance

Growth performance was vital parameter for assessing effects of stocking density on farmed fish or shrimp. Under excessive stocking density, growth performance of aquatic animal is inhibited, which may reduce economic benefits of farming. In this study, *C. quadricarinatus* in the HD group exhibited a lower growth performance than that in the LD group as evidenced by decreased body weight, SGR and WGR. The growth performance was inhibited when the stocking density reached to 207.15 g/m^2^ in the rice-crayfish system after 90 days of farming. The similar results have also reported in other shrimp species. For example, the growth rate of white shrimp (*Penaeus vannamei*) reared at high density (180 shrimp/m^2^) was significantly lower than that at low density (90 shrimp/m^2^) at the 30th week (Araneda et al., 2008). The black tiger shrimp (*Penaeus monodon*) under low density (400 shrimp/m^3^) had a higher average daily weight gain, SGR, and final biomass than that at middle density (450 shrimp/m^3^) and high density (500 shrimp/m^3^), while the FCR (food conversion ratio) was lower at low density after 127 days (AftabUddin et al., 2020). There are many reasons for the poor growth performance at high density, such as the availability of food, water quality, habitable space and physiological status (Riar et al., 2021). A study on *P. monodon* has demonstrated that negative effects of stocking density on growth performance may be attributed to deterioration of water quality (Nga et al., 2005). Irwin et al. (1999) suggested that the turbot reared at high density had lower growth rate due to the increase of social interactions. In this work, the water quality parameters were similar in the three groups and met the *C. quadricarinatus* farming standard (Pan et al., 2017) because of the purification of rice fields. Thus, the inhibition of growth performance was more likely due to the interspecific competition. In the integrated rice–crayfish farming system, the shallow water and rice cropping limited the survival space, which may lead to intensification of interspecific competition under high stocking density. A considerable amount of energy was consumed to cope with intensive social interaction, resulting in the reduction of energy used for growth and metabolism activity (Liu et al., 2016; Wang et al., 2022b).

### Effect of stocking density on antioxidative status

The antioxidant defense system is crucial to the stress response mechanism (Jiang et al., 2014). It has been proven that the activities of antioxidant enzymes can be regarded as indicators of oxidative stress (Wang et al., 2013). Various antioxidant enzymes and non-enzymatic antioxidants can effectively remove excess reactive oxygen species (ROS) and protect against oxidative damage (Zhang et al., 2022). However, when the stress exceeds the tolerance, the scavenging capacity of the antioxidant defense system is decreased (Winston, 1991; Mathew et al., 2007). In aquatic animals, high stocking density can cause oxidative stress and change antioxidative status, which is related to species and farming conditions (Liu et al., 2017; Dorothy et al., 2021; Campa-Córdova et al., 2022). The antioxidant parameters, such as SOD, CAT, GPx, GSH and T-AOC, were inhibited by high stocking density in largemouth bass (*Micropterus salmoides*) (Kommaly et al., 2021) and tiger puffer (*Takifugu rubripes*) (Zhang et al., 2019), which may cause an oxidative damage. However, the study on *L. vannamei* reared in biofloc system showed that the activities of antioxidant enzymes (SOD, CAT, and GPX) were increased with increasing stocking density (Dorothy et al., 2021). Similarly, the data in olive flounder (*Paralichthys olivaceus*) indicated that high stocking density significantly increased the expression and activities of SOD and CAT (Choi et al., 2019). In this study, the levels of SOD, GPx, GSH and T-AOC increased in the HD group after 90 days farming, indicating antioxidant defense system was activated to copy with the adverse stimulation caused by high stocking density. In addition, the activity of CAT in the HD group was significantly lower than that in the LD group, indicating chronic oxidative stress may depress the activity of CAT. This was probably because the CAT consumption was greater than its synthesis under chronic oxidative stress caused by high stocking density.

Under oxidative stress, the excess ROS attacks lipids, inducing lipid peroxidation (Yin et al., 2011). MDA is a final product of lipid peroxidation (Wang et al., 2022a). Hence, it is generally recognized that the content of MDA can reflect the level of oxidative damage in organisms (Han et al., 2022). It has been reported that the content of MDA significantly increased with the increasing stocking density in many aquatic animals, such as *M. salmoides* (Jia et al., 2022), *S. maximu* (Jia et al., 2016), and blunt snout bream (*Megalobrama amblycephala*) (Wang et al., 2018). In this study, the significant increase of MDA content in the HD group also proved that long-term high stocking density caused oxidative stress.

### Effect of stocking density on mitophagy

The liver is a highly dynamic metabolic organ and a major site of protein synthesis, lipid metabolism and detoxification (Qian et al., 2021). These metabolic processes require a high amount of energy. Mitochondria is the primary energy-generating organelle and its function readily deteriorates under stress condition. To maintain mitochondrial quality, cells can invoke a mitochondria-specific form of degradative process, named mitophagy, to remove damaged and dysfunctional mitochondria (Saito et al., 2021). Mitophagy shares some key regulatory proteins, such as LC3 and p62, with macroautophagy, and it is also regulated by specific proteins including PTEN-induced putative kinase 1 (PINK1), Bnip3, and Fundc1 (Urbina-Varela et al., 2020). The mitophagy is susceptible to external stress, such as hypoxia and nutrient deprivation, which, in turn, significantly influences mitochondrial function, cell survival and energy homeostasis (Ke, 2020). It has been reported that mitophagy is triggered to scavenge impaired mitochondria and reduce ROS level under oxidative stress (Fan et al., 2019a). Thus, mitophagy is considered as a protective response against oxidative damage induced by adverse stimuli (Fan et al., 2019b; Garza-Lombo et al., 2020). In aquatic animals, lots of exogenous stimuli, such as starvation, hypoxia, and bacterial or viral infections, have been proven to cause the activation of autophagy. For example, copper exposure upregulated the mRNA levels of LC3a, PINK1, Parkin, and induced mitophagy in *L. crocea* (Pan et al., 2020). Dietary methionine deficiency led to induction of mitophagy *via* PINK1/PARKIN axis in the liver of rainbow trout (*Oncorhynchus mykiss*) (Seite et al., 2018). In this study, the KEGG analysis showed that the mitophagy pathway was activated under high stocking density. It is worth noting that the key genes including *lc3a, p62, bnip3,* and *fundc1* were significantly upregulated in HD group. We speculated that high stocking density is a stressor that could induce ROS production and further trigger mitophagy. The activation of mitophagy is an adaptive response which may alleviate oxidative damage (Tian et al., 2022). Similar data were also reported in previous study, where mitophagy was activated to maintain cellular homeostasis in loach fin cells under oxidative stress induced by doxycycline exposure (Shan et al., 2022).

### Effect of stocking density on endocytosis

Endocytosis is an essential and highly dynamic biological process that is responsible for the internalization of transmembrane receptor ligand complexes, lipids and pathogens (Schroeder and McNiven, 2014). It plays an important role in maintaining cellular homeostasis and interacting with environments (Mellman, 1996), which is mainly manifested in controlling the composition of plasma membrane (Doherty and McMahon, 2009), participating in immune regulation (Lv et al., 2021), absorption of nutrients and other signal transduction of physiological activities (Leborgne-Castel and Luu, 2009). It has been reported that environmental stress-induced adaptive programs can regulate bulk endocytic flux and alter endocytosis (Shin et al., 2021). Increased endocytosis may strengthen cellular resilience *via* elevating nutrient intake under stress condition (Sébastien, 2021). In addition, the alteration of endocytosis during stress may counteract deleterious effects through switching on or amplifying specific cellular pathways (Lopez-Hernandez et al., 2020). Previous studies also suggested that the significant change of endocytosis pathway could be induced by adverse environmental conditions in aquatic animals, such as nitrite (Yu et al., 2019), salinity (Qin et al., 2020), copper exposure (Xing et al., 2021) and cadmium exposure (Zhang et al., 2021). Vega et al. (2010) suggested the upregulated endocytosis played an important role in the recovery from damage under stress conditions. In the present study, we found that high stocking density induced the upregulation of endocytosis in hepatopancreas of *C. quadricarinatus*, which probably was a result of oxidative stress. The increased endocytosis may be an adaptive response which enhanced nutrients uptake to maintain the cellular homeostasis.

### Effect of stocking density on metabolism

Dynamic change in metabolic function is a major mechanism responded to external stress in aquatic animals. High stocking density as a stressor has been confirmed to induce a change in amino acid carbohydrate and triglyceride metabolism in liver of Patagonian blennie (*Eleginops maclovinus*) (Oyarzún et al., 2020). Under a high stocking density, glucose metabolic enzymes were activated, which enhanced energy production to resist the environment stimuli in abalone (*Haliotis discus hannai*) (Gao et al., 2018). A transcriptomic analysis of *M. salmoides* indicated that high stocking density caused abnormal lipid metabolism (Jia et al., 2022). In line with previous studies, our data also showed that the metabolic function of hepatopancreas, such as organonitrogen compound catabolic process, proteolysis involved in cellular protein catabolic process, modification-dependent protein catabolic process and cellular protein catabolic process, was significantly influenced by high stocking density. We suspected the changes of metabolic function may produce more energy to adapt to the stress condition, meaning that less energy and matter were used for growth under high stocking density.

It has been reported that hepatic lipid metabolism is highly susceptible to adverse stress (Guo et al., 2022). In aquatic animals, high stocking density-induced changes of lipid metabolism has been widely reported. The lipid contents were reduced in liver of gilthead seabream (*Sparus aurata*) and piabanha (*Brycon insignis*) under high stocking density, which may reflect a higher lipid utilization to cope with the stress (Montero et al., 2001; Tolussi et al., 2010). Multiple omics analysis showed that high density caused abnormal lipid metabolism in grass carp (*Ctenopharyngodon Idella*) and lenok (*Brachymystax lenok*) (Liu Y et al., 2019; He et al., 2021). Similarly, our data exhibited altered lipid metabolism in hepatopancreas of *C. quadricarinatus* under high stocking density. Meanwhile, the four key pathways associated with lipid metabolism, including fatty acid biosynthesis, biosynthesis of unsaturated fatty acids, glycerolipid metabolism and glycerophospholipid metabolism, displayed an upregulated tendency in the HD group, which may be an adaptive response to stressful condition. The results were consistent with previous studies. For example, activated biosynthesis of unsaturated fatty acids can maintain the membrane fluidity, regulate physiological state and provide energy under starvation and hypoxia stress (Jiao et al., 2020; Ma et al., 2021); Glycerolipid metabolism had an active effect on response to thermal stress in the liver of *S. maximus* (Zhao et al., 2021); yeast (*Saccharomyces cerevisiae*) maintained the membrane homeostasis by activating glycerophospholipid metabolism (Xia et al., 2019).

## Conculsion

This study showed that *C. quadricarinatus* is suitable for growing in integrated rice-crayfish farming system according to the growth performance and survival rate. However, the growth performance of *C. quadricarinatus* was significantly inhibited when the stocking density reached up to 207.15 g/m^2^ (the stocking density of the HD group on 90th day). Meanwhile, the high stocking density caused oxidative stress after 90 days of farming. In order to cope with the adverse change of physiological state, the endocytosis, autophagy and lipid metabolism pathways were activated in the hepatopancreas of *C. quadricarinatus*, which may maintain cellular homeostasis, strengthen cellular resilience and provide energy. In addition, the activation of these pathways consumed a considerable amount of energy, resulting in the reduction of energy used for growth activity, which may be a potential mechanism to explain the inhibition of growth under high stocking density. In summary, our study provided a reference for optimizing the stocking density of *C. quadricarinatus* in an integrated rice-crayfish farming system.

## Data Availability

The datasets presented in this study can be found in online repositories. The name of the repository and accession number can be found below: NCBI; PRJNA884003.
