# Supplementary material for: Effects of stocking density on the growth performance, mitophagy, endocytosis and metabolism of Cherax quadricarinatus in integrated rice–crayfish farming systems
Source: Front Physiol. 2022 Nov 28;13:1040712. doi: 10.3389/fphys.2022.1040712 (PMC9742548; doi:10.3389/fphys.2022.1040712)
Supplement: Supplementary file 1 [file Table1.DOCX]

**Table S1. Specific primer sequences for qPCR in the study**

| **Type** | **Gene** | **Primer sequence (5’-3’)** |
| --- | --- | --- |
| Mitophagy-related genes | casein kinase II subunit alpha (*csnk2a1*） | F: ATAGATTGGGGGTTGGCGGA  R: TGAAGTATCTGGAGGCCACTCT |
|  | sequestosome-1 (*p62*) | F: AGGCCCCAAGGAGGTAATGA  R: GGGGTGACTTGGGTTTGGTA |
|  | Bcl-2 nineteen kilodalton interacting protein 3 (*bnip3*) | F: GAGACTGTGCTGAAAGGCGT  R: AGTTGCCCACCATAACACGG |
|  | autophagy-related gene 9A (*atg9a*) | F: ACCTCAACCAACCAGACAGC  R: AAGGCCATCACACGACAGAG |
|  | TBC1 domain family member 15-like isoform X2 (*tbc1d1*) | F: GTGTTCCGATGATGAGGCCA  R: TGCAATCCTGTAGCTGCGAA |
|  | microtubule-associated proteins 1A/1B light chain 3A (*lc3a*) | F: TCCTCCTCATCAACCAGCGT  R: CATGTAGAGGAACCCGTCTTCG |
|  | FUN14 domain-containing protein 1 (*fundc1*) | F: CGCTGTTGGTGGTTTGTCAG  R: CACCGCCTCATCTGTAGCTC |
| Endocytosis-related genes | heat shock protein 70 (*hsp70*) | F: GGGTATTGAGACAGCCGGAG  R: TCATAGCACGTTCGCCTTCA |
|  | clathrin light chain (*clta*) | F: TAAGACAGGAACAGCACCCG  R: CCGTGAAAGAGAAAGCCCCA |
|  | ras-related protein Rab-5C (*rab5c*) | F: ATTACCGTGGTGCTCAGGC  R: GATGCCTGCCTTTGGAGTTC |
|  | ras-related protein Rab-7A (*rab7a*) | F: TTTGGGATACAGCCGGTCAA  R: CTCGCTTTGTTGATACCGCC |
|  | ras-related protein Rab-11A-like protein (*rab11a*) | F: GTCAGCAGTAGGGAGAGAGC  R: TTGTTGGAGGAAGATCCGCTA |
|  | suppressor protein of bem1/bed5 double mutants (*vps4*) | F: AGACAGACGACGCACATTCA  R: CATGAAGACCGGCCACATCT |
|  | sorting nexin-12 (*snx12*) | F: GCCTCGTCTCCAAGAAACAA  R: AACCACAATCTTGCTGTCCCT |
|  | actin-related protein 2/3 complex subunit 4 (*arpc4*) | F: TGTTCGGCACACACTCTCAG  R: TCCTGATGGAGTTGATGGATGG |
|  | adaptor protein 2 complex subunit mu (*ap-2*) | F: ATTTCCCTGCCATTCCGAGT  R: GCTGAACCCCCGACGTATTA |
|  | dynamin superfamily protein (*dnm*) | F: ACGTGTTCTCAATCAGCAGC  R: GCTGACCCTGATCCCTCAAT |
|  | ras-like GTP-binding protein (*rho1*) | F: TGATAGACTGCGACCCCTCT  R: ACTGGCTCTTGTTTCATCTTCTGA |
| Reference gene | *β-actin* | F: ATCACTGCTCTGGCTCCTGCTACC  R: CGGACTCGTCGTACTCCTCCTTGG  GenBank: AY430093 |

Gene sequences applied in this study

Sequence No.1 *csnk2a1*

CTGGCTGGGTTTGGTCGGACGGTTTACCGGGGGCTCCACAAGATGCCGTTGGCAAGCCGTGCTCGGGTTTATGCAGATGTCAATGCCCACAGACCACATGAATATTGGGATTATGAATCTCATGTTATAGAATGGGGGCAACAAGATGATTATCAACTCGTGAGAAAACTTGGCCGAGGCAAATACTCTGAAGTGTTCGAGGCTGTAAACATCAACAACAATGAAAAATGTGTTGTCAAGATTCTCAAGCCTGTCAAGAAGAAGAAGATTAAGAGAGAAATTAAGATACTGGAAAATCTGCGAGGTGGGACCAACATCATCACACTCCAGGCTGTCGTGAAAGATCCAGTTTCTCGCACACCAGCATTGGTGTTTGAACATGTTAACAACACTGATTTCAAACAGCTATACCAGACGCTTAATGACTATGATATTAGATACTATCTCTATGAGCTTATAAAGGCTCTAGACTATTGTCACAGTATGGGAATAATGCATCGAGATGTTAAACCCCACAATGTCATGATTGATCATGAACATCGGAAATTACGACTCATAGATTGGGGGTTGGCGGAATTTTATCACCCAGGACAAGAGTACAATGTTAGAGTGGCCTCCAGATACTTCAAGGGTCCAGAACTACTCGTAGATTACCAGATGTATGATTACTCTCTTGATATGTGGTCATTGGGTTGTATGCTGGCGAGTATGATATTCCGTAAAGAACCCTTCTTCCACGGTCACGACAATTATGACCAGTTGGTACGCATTGCCAAGGTGTTGGGCACTGAAGAGTTGTATGAATATGTTGAGAAATATCAAATTGAGCTAGATCCTAGGTTCAATGATATTCTTGGAAGGCATTCACGAAAGCGGTGGGAAAGGTTTGTTCATAGTGAGAATCAGCACTTAGTATCGCCAGAAGCACTTGACTTTTTGGACAAATTACTACGCTATGACCACCAAGAGCGCCTCACGGCTCATGAAGCTATGGAACATCCATATTTTTATCCCATTGTAAAGGAACAAGGTCGCCACATGAGCTCACCAACACCTGCTGCTCCTGCTTTAACTGGGGTCTCGGAA

Sequence No.2 *p62*

CAAATGGCCTCAGAACATGTTGCCAATGTAATGCGTGGTATCTGGACTGCTTGGACTGGTCATCAGGGCCCTGGCCAACAGGGACCTGGTCTTAAAGCATCTTCAAGTTCTTCTTCTTCTTCAAGCTCTTCATCATCAAGCTCTAGTGGAGATTCCCCACAGCAAAAATCAAAAGAGCCCAAGGGACGTGAAGGTCAGCAGCAGAAAGACGTTAGCATGGAAGCTAATCAGACTACTGGTGAAGAGTACCTGAGGAATGTTGGCGAGACTGTTGCAGCAATGTTGGATCCTATTGGCATTGATGTCGAAGTGTCTGTAGAGCACAATGGAATTCGCCAGCGCTGCAGTCTAAGCAAGGATGATGTAGAGAATCGTAAGACTCCAACAGTTTTGTTCCCTCCAAGTCCTCCAAAGGAACCAGAGTCACCTGAAAAGTCTTTGGCATCGGAAACTAGTGCAAATATGGAAGTACAGACCGATAGTCCAAGGCCCCAAGGAGGTAATGATGGTGGGGCAGAAATAATGGAAGTAAGCAGCCCTGAAGCAGGACCAAGCCAAAATGCTGGAGATCAAATGGAAGGAGAGCATTCTTCAGACATAGAGGACTGGACACTGGTTAATCAGGATGCCAATTCAGAATCCCAGGGTGGTACCAAACCCAAGTCACCCCCCACTGAAAGGAGAGTTGTTTATCCAGATTTGGCAGTGATTGAAACTCATCCAAATCCTGTCATTCAGCGTGCTCTGGAGCAGATGCAGGCTATGGGCTACAACAATGAGGGTGGATGGCTCACCAACTTGCTTGAAATGAAACAGGGAGACATTAACCAAGTGCTTGACCTGCTCCAGCCAGTCAACAAG

Sequence No.3 *bnip3*

CCCGAGGACTTCGGCAACGAGACGGACTCTGACATAGAGGTCTTGGCTCAGTCAGAGTCATGGGTGGATCTGTCCAACCAGCCGGGTAGTCCTGACCGGGTGACACCATTGCCCTTTGGGAATGGTGAGGAGTACTTGCGACTGTTGCGAGAGGCCCAGAGAGAGAGCAACCAGTCTTCAGAGCGAGTGTCATTAGCTTCCTCTCGTAGGGACACACCTAGGGACTCTCCACATGATTCGCCAAAGTCCCCACCAAACAGCCCTAACACTGAGATGGCCACCGACCCAGAGGAGACTGTGCTGAAAGGCGTATACATTAATTACTATAATAAGGAAGGAGACTTTATTCGGGTTGAGAAGAACACAGAGACGGATTGGATCTGGGACTGGAGTAGTCGGCCTGACCAGACTCCACCAAAGGAATGGCGGTTTAGTCATCCTCGTAAAGGTGTGAGCCGTGGTGCCAGTATCCGCCGTGTTATGGTGGGCAACTCTTCACTCTTCTCTCGTGATGTCCTTTACACACTCCTTATCACCAATGTTTTATCGCTTCTTCTTGGTACTGGCATTGGGATCTGGTTGTCGAAACGATCAGGTGGTGAAGTTGTGATGACTTTACCCATCAAC

Sequence No.4 *atg9a*

ATGTCTCAGAATTTCCAGACAAGCTACCAGCCCTTAGGAGGGGCGAGTGACAGAGATGGAATTTCTAGAGGAGAGGATGGGGAGAGAGATGATGATCTCTCCCCAGATACACACACCAGTATGGTATTTCACGTTGTTCCCAAGTCCAGCAAGTCAAGGTGGAACCACCTGGAAGATTTGGATTCCTTCTTCACACGGGTGTATCAATATCATCAAAACCATGGGATGCTGTGTATGATGCTGCAACAGTTATTCGAACTTGTTCAGTTTATATTTGTAGTTGTATTCACCTCCTTCCTGTTTACTTGTGTAGACTACGATATTCTATTCAGAAACAGATCTCCAAAAAATTTTAATGCGACAGACAAAATAACACTGCTTGATGCTATACGCACTTGTGATGAATGTTTGGAACACTTCAACTGGTTATTGGTTTTCATCATTGTACTATCAGTGGTAATCTGGCTGCTTCGTCTTGTCCACGCTGGCTTCACCCTCTTCCAATTCTGGGAAATCAAGCTCTTCTTCAACAAGGCACTGGGCATCCACGACAATGACCTTGGCAATATTACATGGGAAGAAGTGGAGCAGAGAGTGATGGAAGTGCAGGTTGAGCAGCGTATGTGTATCCACAAGGAAGTTTTGACAGAGTTGGATATATACCATAGAATTCTTCGCTTCACCAACTACTTTAATGCAATGGTCAACAAAGAAATAATTCCACTTAAGTTTGAGATGCCCTTCTCTGGAGAAATGGTCTTTCTCACAAAAGGCCTGCGCTACAACATAGAAATGCTACTTTTTTGGGGACCATGGGCACCATTTAAGAACAACTGGCACTTAAGTGATGAATATAAGAGACTGGAGAAACGACAAGAGCTGGCAGAGGAACTTAGTAAAAGATTTCTCTATGTTGGCATTGCAAATCTTGTCCTCTCACCTTTAATATTTATTTTCCAGTTCCTCTATTCTCTCTTCAATTATGCAGAAATGATAAAACGTGAACCTGGTAGCTTGGGAAGTCGTCGTTGGTCTCATTATGGGGAAGTGTACCTACGGCATTTTAATGAACTTGATCATGAGATGACAGCACGTCTTAACAGGGGCTATAAAGCTGCATCACAATACATGACAATTTTTTCTTCTCCTATCATGGCTGTAGTTGCTAAACATGTTGCATTTGCATGTGGGGCATTATTTGTTGTCTTGGCAGGTTTGAGCATATATGATGAGGATGTTTTACAAGTTGAACATGTGTTGACATTTATGACTATTTTAGGGGCAGTTCTTGCTGGATGTCGTGGCTTTATCCCTGCTGAAAATCTGGTATTCTGCCCAGAACTGTTATTGAGGAGGGTTTTGGCAGAGGTGCATTACCTCCCAGATCACTGGAGAGAAAAGGCTCATACATCCAGGGTTCGTCAGGAGTTCTCTCAACTATTCCAGTACAAAGCTGTGCACATAGTGGAAGAATTAGTGTCACCCATAATTACCCCTCTCATTCTCATCTTCAGGCTGCGTTTCAAAGCTTTAGACATTGTAGATTTCTATCGCAATTTTACAGTAGAAGTTGTTGGGGTTGGTGATGTTTGCTCCTTCGCCCAAATGGACGTTAGAAGACATGGTAGTCCAACATGGCAGCCAGATGTTCTGTCAGAGGACCTCAACCAACCAGACAGCTCAAGTAAACCTGGTATGACGACAGATGGTGGCAAAACTGAGCTTTCTCTCATTCACTTCACAATGACAAATCCCAAATGGAGACCTCCAAAAGAATCCTCTGCTTTTATTACTGCTCTAAAAACACAAGTTCAGAGAGATATGAATGCTCTAACAACACTACAGGAAGAAAATGCTCTCTTCTCTTCCTTGAACTCTCTGTCGTGTGATGGCCTTGGTATGGTAGCTACTAGCATGTTCCGCCCTCCCTGGATGATGGGTGGACCTCAGATTGGTGGGCAGCTTCAACAATCCTACTGGCCACCCATAGGTCTGCGGGGTAGGATTTCACGTGTTGAAGGTTCCCGCAATGGTCCCAGTGGTGGAATATTATCATCCATACAGCAAATGGGATCAAGTGTAGAGGGTCCACTCGGGACAAGTACCATGACTTCAGGTCCCATTATTGATCCAGTTCTTGCCCCTAGTACTATTGGCCCTTCATCAATTATTGGTCGAAGTTTAATGGCGCCACTTCCTCAGGATCTCACTGCTGTAAACATGAGTCTCAGTACATTGTATCTGCATGAGCTGCATCAGCGACACCGCCACCGGGCCATGTACACTGACACCCCACACAATCCTTCAGTATCCCAGGGTCCTCAAGCTGGAGTTTTCCACTCTGTTCCTTTCCAGGGCAGGCCTCCTCCAGACAGTGAAGCTGCACCTTCAGTGTCATTCCAGCGTGCACAAGAGAAGACTCCATTGTTAGCTGACCCT

Sequence No.5 *tbc1d15*

ATGGACGAAGAACTGCTTGTGTACGAGCAGGACGAGGTCTATACTCTGCCCACATCACAAGAAGAGCTATCAGTTAGTGGAAAACTGCGCATCATAGAAAGACGGCATGGAGTTGTTGTTGAGTGGCGGTGTTCCGATGATGAGGCCACAACAGATAGTGAATGGGCAGTTATCAACTCTGCTGCTGTTACATTTACACACCACAATGTCTCAGATTCAGTGGAGATAAGCACACCAAGACGCAATGTTCGCCCCATAGCCATCGAACTAGTTGATCTTCGCAGCTACAGGATTGCAGATGATGATTCCCTTGTTCTGATTCAAAGAGATGGCACCACTCACCCGGCTCTGGCTTTCCGCTCAGGATCTGTCTTATGTTTCGTTGAAGTCCTCCTCAGATACGTTGCTGTTAAAAGGTCAGAAAAGGATAGCAATTTGTATTTGGTGAGTGACAAGCGACAGGCAGCTATGGATGAGGAGCTTTCAGCCATGAACTTGGTGCCTCCTCGCCGTGGGTTTCCAGGAGCCGGAGGTCCCAACCACTGGCTCAACACACAACACCGGGTATGGGGCTTCATCAACAATTTAAAGCGAGACCCTTATACAACTACTGTTGGAGCACTCTCCAAGTTTTATGATGCTGTTTTATACACTGGAGTGGAGGGTTACATTCGTGAGAGTGATGGAGCAGAGGATGATGTAGCAGAGCTCTTCCACAGTCTTAATGGTTTTGAGGACCTAGAGCCAGGATATGAGCTTCTGACAAAGAAGGGCACCCTTGCTGAGCGTCCGGATGTAGACAGATTAGCGCCTCTCACACATGATGATTTCCAGCAGTTTCTGGATGACGACGGTCGCATCCTGGATGTTGAACCTCTCAAGCAACGAATATTTAAAGGGGGGATCCATCCAAACCTTCGCCGTGAACTATGGTGCTTTTTGCTGGGTCACAATCTGTGGAACTCCACGTACATCCAGCGAGAAGAGAGACGCAGACAGAGAGAGAACCTTTACTTTACTATGAAGCAGCAGTGGAAGACCATTACCGAGGATCAAGAAAGTCGTTTTACTGGGTTCAAAGAACGACGGAGTCTTATTGAGAAAGATGTTAATCGCACAGATCGAAATCATCCATTTTTTGAAGGCGAGCACAATCCCAATGTAGTTCTACTTCACGATGTTCTCATGACATATGTGATGTACAATTTTGACTTGGGCTACGTCCAGGGCATGAGTGACCTTCTTGCTCCCATTCTCTATGTAACACAGAATGAAGCAACAGCATTCTGGTGTTTTGTTGGTTACATGGATATTGTGTACAGGAATTTTGATATGGATCAGTCAGGGATGAAGCAACAACTGAAGGATGTGCACCAAATTGTGCACTTGGTCGACCCAGAGCTCATGTCATACCTAGAAGTGCGTGACTCTGCCAACTTCTACTTCTGCTTCCGGTGGCTTCTTGTTCGATTTAAGAGGGAACTTAATTATGCAAGCACATTACGGTTATGGGAAGTTTTGTGGACTGGTCATCCATGTCCAAACTTCCATCTGTTGGTGGCAGTTGCTCTTCTTGACACCGAGAAGAACACAATCATGGAGAACAAATTTGGATTTACTGAAATTTTAAAACATATCAATGACATTTCGTTACGAATTGACTTGGATGCCACCTTGAAGAAAGCAGAAGCAATATACTTGCAGATTGCATCATCACCCAATGTGCCAGACTGTGTGAGGACAGTGTTAGGTCTTCCTTTACAAGAGCCTTCT

Sequence No.6 *lc3a*

ATGAACACTCAAGCCAAACCTTTCAGGGAAAGGAGAAATTTCGCCCAGCGTCAGAGGGATGTTGAACAGATTAGAGAACAACACCCAAATAAAGTTCCAGTGATAATAGAACGATACCCTGGCGAGAGGCATCTCCCTCTTCTGGATAAAACAAAATTCCTTGTTCCAGATCATGTTACCATGGGAGAACTTGTCAAGATTATAAGGCGGCGGCTACAGCTGCACCCTACTCAAGCATTTTTCCTCCTCATCAACCAGCGTGCCCTTGCCAATGTCTCCAATACTCTTGCTCAGGTGTACGAGCACCACCGCCACGAAGACGGGTTCCTCTACATGGTGTATGCTTCCCAAGAGGTATTTGGA

Sequence No.7 *fundc1*

ATGGGAGGAAGTGGCCAGGCTAAACTGAAAAAGCCAAACGAAGAGGAGGAGTTTGAGGTCCTTGATGTAAGAGGAACTGCTCGTAACGGGATGTCGTGGCTCCAAGAGTTTATGCATGATTTGACCCAGCAGCCTGTCACAAAGCAAGTCGCTGTTGGTGGTTTGTCAGGATGGGTTGTAGGATACTTATCAATGAAGGTGGGCAAAGTTGCTGCAACAGTAGTTGGTGGAAGCCTTCTCATCATGCAACTGGCTGCCCATAAAGGATATATAAAAGTGGATTGGAATAAAGTGAACAGGGAACTTGAGAAGAATGCTAAGAAGCTGAAGCAAGAGGTAGAGTCCACTGTGAATGCTGGAGCTACAGATGAGGCGGTGCGCTTTGCCCGCGAGAACATAGTGTTGGCCACAAGCTTTGGTGGAGGAGTGCTGTTGGGTATTGCTTCTTCC

Sequence No.8 *hsp70*

GCTCTGTCAATTGGTATCGACCTGGGCACTACTTACTCCTGTGTAGGAGTGTTCCAGCAGGGCAAAGTGGAAATCATCGCCAACGACCAGGGCAACAGAACCACTCCATCCTATGTCGCTTTCACTGACACAGAACGACTCATTGGTGATGCAGCTAAAAACCAAGAAGCCCTCAATCCCAATAATACAATTTTTGATGCAAAACGACTGATTGGTCGTAAGTTTAATGACCCCACTGTTCAGACTGACAAGAAACACTGGCCTTTTGAGGTAGTCAACGACAATGGTAAACCCAAGATACGAGTTGAATATAAGGGAGAGAGCAAGAGCTTCAACCCAGAGGAGATCTCGTCCATGGTGTTGACCAAGATGAAGGAGACAGCAGAGGCGTACCTGGGCCAGAAAGTGAAGGATGCTGTCATCACAGTGCCTGCTTACTTCAATGACTCGCAGCGCCAGGCCACCAAAGATGCTGGTGCCATCGCTGGACTCAATGTTCTGCGCATCATCAACGAACCCACAGCAGCAGCAATTGCCTATGGCCTTGACAAGAAAGTGGTCGCAGGAAGCTCAAGAGAACGTAACGTACTCATCTTTGATCTGGGCGGTGGAACATTTGATGTGTCCGTTTTGAGCATCGACGATGGTATATTTGAGGTTAAGTCCACGGCTGGAGATACTCATCTCGGTGGCGAAGATTTTGACAACCGTATGGTCAACCACTTTATACAAGAGTTTCAGAGGAAATACAAGAAAGATTTGAGCAGCAATAAACGTGCACTGCGACGCTTGAGAACGGCTTGTGAACGAGCTAAACGAACTCTCTCGTCCTCCACACAAGCCACCGTGGAGATTGACTCACTACATGAGGGCATCGACTTCTACTCGTCCATTACTCGAGCTAGGTTTGAGGAGTTGTGTTCAGACCTGTTCCGCAACACATTGCTTCCTGTAGAAAAGGCTCTGAGGGACGCCAAGATGGACAAGAGTCAAATTCACGATATTGTGCTGGTGGGTGGATCTACTCGTATCCCCAAAGTGCAGAAGATGCTGCAAGATTTCTTTAATGGCAAAGACCTCAATAAATCCATCAACCCCGACGAGGCGGTGGCCTATGGTGCTGCTGTTCAGGGTGCCATCCTTACGGGTGACCAGTCGGATGGAGTCAAGGATCTGCTGCTTCTGGATGTGGCACCTTTGTCTTTGGGTATTGAGACAGCCGGAGGCGTCATGACTGCTCTCATCAAACGCAACACTACCATCCCCACCAAGCATTCTCAGATCTTCACAACATATGCAGACAACCAGCCGGGTGTCCTTATCCAAGTTTATGAAGGCGAACGTGCTATGACCGAGGATAACAATCTGCTGGGTAAGTTTGATCTCTCGGGTATTCCCCCTGCCCCAAGAGGAGTCCCTCAGATAGAGGTCACTTTTGACATCGATGCCAACGGTATCCTTAATGTGTCTGCCATTGACAAGTCGACAGGCAAACAAAACAAGATCACTATCACTAACGATAAGGGCCGTCTCAGCAAGGAAGAAATTGAGAGAATGGTTCATGAAGCAGAGAAATATTCCGAGGAAGACGGTCGTCAGCGAGAGAGAATCGAGAGTAAGAATCGTCTGGAGTCTCTGTGTCTCGGCCTGAAGAACAGCCTGCATGAAGAAGGTGTCTCCAGTAAACTAACGGAGGAAGAGAAAAAGAATATCTCCAAGAAAGTGGAGGAAACACTCTCCTGGATTGACGACAACCAGTTGGCAAACAAGGAAGAGTATGAGTTCAAAGCCAAGACTCTAGAAGACCAGTGGAAACCTTTGACTGCCAAAATTTATGGCTCTGGAGGTTATGGACAAGCAGGTCAAGGACCAACCGCTTCCAATACCTCCAGTCGACCAGGACCAACTATCGAGGAGGTTGAC

Sequence No.9 *clta*

CCTCTCTCTATCACCATGGATGGGTTTGGCGATAGTTTTGTACCTCTAGTGGAAGGGTCTGCACCAGCAGCAGAGGTGGATCCTGCTGCAGAATTCTTAGCTCGGGAGCAGGACCAGCTAGCTGGCTTGGGGGACGATATTCTTCCAGCTACCCTAGGTCAAGATCAGTCAACAGTGGCATCAGGCCTTGGTGGAGAAGCTGACTTGTTTGGCTGTGCCCCCGTCAATGGTGGCCCTGACCTGGAGAGTTTTGAGATGCTGGGTGGAGATGAGGTTGCCCAAGAAAAAGCTGCTCCTCCTCCTCCTTCAGTCTTTGGTTCTGTGGACTTTCTACCTCAGCCCTCGGGAGAAGATCCCTGGGCCTCTGCACAAGCTCCTTGTGCAACGGATGAGTCTGTTGCTCAACCTGTTGAGGAGACTCTTGGCTTTACTGAATCCCTTTCCTCCCAGTCCTTTACCATTGGTGAAGGAGAGGGGTTCTTACCATCATCTGACCTAGCAGGAGCAGCAGCAGCAGAAGCACCAGCATCTGATGCAGTAACAGCTGAGGTCGCAGCAGCTAAGACAGGAACAGCACCCGAAAGGCGTTATTCAGGCTTTGGGTCTGATTTTGGGGGAGCATCTGTGCCTGCTGGTGATACTGGTACCACTTCTGGGGCTTTCTCTTTCACGGGTGACCCAGCACCACCAGTGACCACGTCTGAACCTGACTTGGCCCGGTCGCCTATTCCCCATGTGGTAAGGGAGGATCCAGAGAAAATAAAAATCTGGCGTGAGCAACAACGGATCCGTCTGGAGCAGAAAGATGCTGCTGAGGAGGTAAGCAAGATAGAGCTTAAGGAGAAAGCAAGGAAAGAGCTTGAAGACTGGTATAAGCAGCATGAAGAACAGGTTGCTAAAACACGACAGGCCAACAGGTCTGCTGAGAAGGAACTAGTTGCTGATACAGCGAAGATGGAACCAGGCACAGAGTGGGAACGAATAACCAAGTTGTGCAATTTTAACCCTAAGACTTCCAAATCCTCAAGAGACATT

Sequence No.10 *rab5c*

ATGGCACAGAGAGGAGGTGCACAACGACCCAGTGGTGGTGGCCAGGGCAAGATCTGTCAATTCAAATTGGTGTTACTGGGTGAATCTGCAGTTGGAAAGTCTTCTCTTGTTTTAAGATTTGTTAAAGGTCAATTCCATGAATACCAAGAATCTACCATTGGTGCGGCCTTCTTGACACAGACAGTATGTTTGGATGATACCACAGTCAAATTTGAGATCTGGGACACAGCTGGACAAGAAAGGTATCATAGTCTAGCTCCTATGTATTACCGTGGTGCTCAGGCAGCAATTGTCGTCTATGATATCACAAATCAGGACACATTTGGCCGAGCCAAGACTTGGGTAAAGGAACTCCAAAGGCAGGCATCCCCCAACATTGTGATTGCTTTAGCAGGCAACAAAGCAGATTTAGCCAATAAAAGGATGGTGGAATATGAGGAGGCTCAAACCTATGCAGAGGAGAACTCCTTGCTTTTTATGGAAACCTCAGCCAAGACTGCTATGAATGTTAATGATATTTTCTTGGCAATAGCTAAGAAATTGCCAAAGAGTGACAGTAATGCCAGTGGTTCTGTCAGTGGTAATGTCAGCCTGTCTAATAACCAGCCAGCGCAGGGTACTGCTGGCTGTTGCAAG

Sequence No.11 *rab7a*

ATGGCATCTCGCAAGAAGATTCTTTTGAAGGTCATTATCCTGGGTGATTCGGGGGTTGGGAAAACATCTCTTATGAACCAGTTTGTCAACAAGAAGTTTAGTAATCAGTACAAGGCCACCATTGGTGCAGATTTTCTAACGAAGGAAGTGATGGTGGATGATAGACTTGTCACTATGCAGATTTGGGATACAGCCGGTCAAGAAAGATTCCAGTCACTGGGCGTCGCATTCTATCGTGGAGCTGACTGCTGTGTTTTGTGTTATGATGTCACATCTCCTAATTCCTTTAAATCTTTAGACTCTTGGCGAGATGAATTTCTAATTCAAGCTTCACCACGTGATCCTGATCATTTCCCATTTGTTGTTCTTGGAAATAAGATTGATCTGGAAAATAGGGCGGTATCAACAAAGCGAGCACAGCAGTGGTGTCATAGTAAAAATGAGGTTCCTTATTTTGAGACCAGTGCAAAAGAGGCCATAAATGTTGAGCTGGCATTCCAAACCATTGCCCGAAATGCCCTTGCCCAGGAGTCTGAAGTAGAGCTTTACAATGAGTTTCCTGACCAGATCAAGCTGACCAATGATAACAAGGCTAAGCAAGATGCATGTTCTTGC

Sequence No.12 *rab11a*

ATGGGGAACAGGGACGACGAATATGATTATTTATTCAAAGTGGTGTTAATTGGAGATTCTGGTGTTGGCAAGAGTAATCTTCTTTCAAGATTTACGAGGAATGAATTTAACCTTGAGTCAAAGTCCACTATTGGTGTGGAATTTGCAACGCGCAGCATAGAGGTGGATGGAAAGACCATAAAGGCACAAATTTGGGACACTGCAGGACAGGAGAGGTATCGAGCCATCACGTCAGCCTACTATAGGGGAGCTGTAGGTGCTCTCTTGGTGTATGACATTGCCAAATTGCTCACATATACCAATGTAGAACGTTGGCTGAAAGAACTTAGAGATCATGCTGATCAAAATATTGTCATCATGCTTGTAGGTAACAAATCAGACTTGCGCCATTTACGGTCGGTGCCCACAGAGGAAGCCAAGGCATTTGCCGAGAAAGAGGGATTGTCCTTTATTGAAACTTCAGCGTTGGACTCCACCAATGTTGAGACAGCCTTCCATAACATCCTCACAGAAATTTACAGGATTGTCTCCCAGAAGCAAATTCGTGATCCTCATGACCACGATGACAGTCCAACAGCCGATGTAAAGGCTATTCACGTGGAACCAACTGTAAATGCCGAGAGTGTGCGCAAGCAATGTTGTCAGCAGTAGGGAGAGAGCTCCTCCTCACACCTGGAAATTTGGGCAAAGGAAATCTATCGCATTGTCTCGAGGCAGCAGCTCCCAGACAGCGGAACGAATCAACCTCCTGGCGATAAGTTAATCACTGTGGAGCCCACCGAACTCCCTAGCGGATCTTCCTCCAACAACTGCTGTGCCCGC

Sequence No.13 *vps4*

ATTAATATGAAGTGTACAGATTCAGAAGACACCATGAGTGCAGGACCCAACAACATACAGTTATATCAAGAGGCCTTGTCATGTTTAGAACTCATTGCTTCTCAGTTGGAGAGTAACACAGACTACCCGGTAGTGGATCACCTGTATGAGAAGTGCAGCACTTCCATCACCATCTTACGTACATCCCAGCCCAACCCCCATATATCGGCATCTTTAGAGAATCTTTTAGAAACCTTAAGTTTCCAAAAGTCCTTGTTGGATTCTCGACGTTATGGTTGCAAACCAGACAGACGACGCACATTCAGTGAAGGATCATTTCAGGATTCTGATAACAAAAAGACATGTGGGAACTGCTTGAAACCAAGTAAAAATGAAGTCCAGCAAGAGAGTAACCATAGTCTCACAATAGAGGATATGATATTACCTTGCAATACCAAAGGTGGGTCAATGGAAGATGTGGCCGGTCTTCATGATGTTAAGCAAATATTAAAGGAAGCAGTGATCATGCCCCTGCAATATCCACAGCTCTTTCAAGGTGGAGCAAAGCCCTGGACTCGTTTATTGCTTTATGGCCCCCCAGGCACTGGAAAAACCAAACTAGCTCGAGCTCTGGCCACAGAACTTTGCTGTCCATTTTATTGTGTTTCATCTGCCAGTCTCTTCTCTTCTTGGGTTGGAGAATCTGAAAAATTAATCAGAGACTTGTTCCATCATGCAAAGCAGCAAGAAGGCCAGAGTATTATTTTCATGGATGAAATTGACAGTTTGTGTAGAAAACGTTTACCAACAGAAGATGAACACTCAAGAAGAGTGAAGTCGGAGCTGTTGCGGCAAATTGAGGGAGTAGAGGAGAGTGAGACTTCAGGTGTGTTCTTGTTAGGGGCCACCAATTGTCCATGGGATCTTGACCCTGCCTTCCTCAGACGCTTCCAGCGACGCATATTTATTCCTCTTCCTGACAGGGAAGGTCGCCGCACTATAATTTCCAGTCAGTTTGGTAGAGTACCATTACACTTAACTGACTCTGAATGGCAGACACTCTTGGACTCTACTGAAGGCTACTCAGGTGCTGATCTTACTCACCTGACTATGGCTGCTGCTTTTCAACCAATTAGAGACTTGCATTCTTCTCGCTTCTGGAGATTCACAGATGACAACAAGATTACCCCATGCTCCAGTGATACCCTTGGTGCCATGCAGTATCCCTTGTGCAAGCTACCAGCAGATCAGATTGTTGCTCGTGATGTAGAATTAAGAGACTTCCTAAGGGCCATCCAGACAACAGCAAGGACAGTTTCACCCAAGATTCTCCAGCAGTATCATGATTTCTCTTCAGCT

Sequence No.14 *snx12*

ATGGCTGACATGACCAATGACTCTACACGTCGCCTCGTCTCCAAGAAACAAACTCTAGACGATGCCTACGCTGCTCCAGCAAACTTCCTCGAGATCGACGTAGTAAACCCAATCACTCATGGTGTCGGCAACAAAAGATATACTGACTATGAAGTTCGGATGCGGACCAACTTACCAGTGTTTAAGGTGAAGGAATCAAGTGTGCGGAGGAGGTACAGTGATTTTGAATGGCTAAGAAATGAACTTGAAAGGGACAGCAAGATTGTGGTTCCACCACTTCCTGGAAAAGCATTAAAGAGGCAGTTACCATTCAGAGGAGATGATGGCATTTATGAAGAAGAATTTATTGAGGATCGACGCAAAGGACTCGAAGTTTTCATTAACAAAATTGCAGGACACCCTCTTGCACAGAATGAAAAGTGCCTACATATGTTCATTCAAGAGCCAGTCATTGACAAAAACTATGTGCCTGGCAAAATTCGCAACACG

Sequence No.15 *arpc4*

ATGTCGGCCACATTACGTCCTTACCTGAATGCTGTTCGGCACACACTCTCAGCTGCAATGTGTCTTCATAATTTTAACTCACAAGTTGTAGAAAGACACAATAAACCTGAAGTAGAGGTTAGATCAAGTAAGGAACTCTTGATGACTGCTGTTGTTGTTAGTCGAAATGAAAAAGAGAAAGTTCTAATAGAACCATCCATCAACTCCATCAGGATTTCCATCTCTATTAAACAAGCAGATGATATTGAACGTATCCTTTGTCATAAATTTATGAGATTTATGATGATGAGAGCTGAAAATTTCATAATTCTACGTAGAAAACCTGTACAGGGCTACGACATATCTTTCCTCATAACGAACTTCCACACAGAGCAGATGTTCAAACACAAACTGGTAGATTTTGTCATTCACTTCATGGAAGAGATTGATAAGGAGATTTCGGAAATGAAACTGGCTGTGAATGCTCGAGCCAGAGAATGTGCCATGGAATACTTAAAAAGATTC

Sequence No.16 *ap2*

ATGTTGGGTGGTTTGTTTATATATAACCATAAGGGGGAGGTGCTTATATCTCGAGTGTACCGGGATGACATTGGACGTAATGCGGTGGATGCATTCAGGGTGAACGTGATCCATGCTCGGCAGCAAGTGCGCTCCCCTGTCACGAACATTGCACGCACCAGTTTCTTCCACATAAAGCGTGCCAACATCTGGGTTGCTGCAGTAACCAAACAAAATGTTAATGCCTCGCAGGTGTTCGAGTTCCTCCTTAAAATGCTGGAGGTGATGAGTAGCTACTTTGGCAAGATATCTGAAGAGAATGTAAAAAACAACTTTGTACTTATCTATGAATTGCTAGATGAGATTCTGGACTTTGGGTATCCTCAGAACACAGACACAGGAGTTCTCAAGACATTCATTACTCAGCAGGGTATTAAGACAGCAAGCAAGGAGGAGCAGTCACAGATCACCTCTCAGGTCACTGGACAGATTGGGTGGCGGAGAGAAGGCATCAAATACAGACGCAATGAACTGTTCCTTGATGTTATTGAATATGTCAATTTGCTGATGTCTCCTCAAGGTCAGGTGTTGTCTGCCCATGTGGCAGGCAAGGTGGTGATGAAAAGTTATCTCTCTGGGATGCCTGAGTGCAAGTTTGGCATCAATGACAAAATTGTTATGGATGCGAAGAGAGGTTCATCAGACGAGTCTCAAAGGTCTGGGAAGACCAGCATTGCCATTGATGACTGTCAATTCCACCAGTGTGTGAAGCTCTCCAAGTTTGAGACTGAACACTCTATTTCATTTGTTCCTCCAGACGGTGAATTTGAGCTTATGAGGTACCGTACAACAAAGGACATTTCCCTGCCATTCCGAGTGATTCCACTAGTACGTGAGGTTGGACGCACAAAAATGGAAGTGAAAGTTGTCATAAAGAGCAATTTTAAGCCCTCACTCTTAGCACAGAAGATTGAGGTGCGGATCCCTACTCCACTTAATACGTCGGGGGTTCAGCTTATATGCATGAAGGGGAAGGCCAAATACAAAGCCTCAGAAAATGCTATTGTGTGGAAGATAAAGCGAATGGGTGGTATGAAAGAATCTCAAATCTCTGCTGAAATAGAATTGCTGCAAACAGACACAAAGAAGAAGTGGACCAGGCCTCCAATCTCAATGAACTTTGAGGTGCCGTTTGCTCCATCTGGCTTCAAGGTTCGCTACCTGAAAGTATTCGAGTCGAAGCTTAACTACTCTGACCATGACGTAATCAAATGGGTTCGTTGCATCGGCCGTTCGGGACTCTACGAGACCAGATGC

Sequence No.17 *dnm*

ATGTCGGGCAACGTGGGGATGGAACAGCTCATCCCCATTGTGAACAAACTACAAGATGCCTTCACACAGTTGGGAGTGCATATGCAGCTGGATTTACCTCAAATTGCTGTAGTTGGTGGACAATCAGCTGGCAAGTCCTCTGTGCTGGAGAATTTTGTGGGACGGGACTTTCTACCTCGTGGTTCTGGTATTGTTACACGACGACCACTAATCCTACAGCTCATTAACCACCCGCATGAATATGGAGAGTTCCTACACAAGAAGGGTGAAAGGTTTACCAGCTTCGATGAAATTCGCAAGGAAATTGAGACTGACACAGATCGCATTACGGGTCAGAACAAAGGCATTTCTCCCTTGCCTATTAACCTGAGAGTTTACTCGCCTAATGTGCTGAACCTGACACTCATCGACTTACCTGGCTTAACAAAGGTACCCATTGGTGACCAGCCACCAGACATTGAGCAGCAGATTCGCAACATGATCATGACGTATATTACCAAAGACAGTTGTCTAATCTTAGCTGTGTCACCTGCTAATTCTGATTTAGCCAACAGTGATGCACTCAAGTTATCCAAAGATGCTGATCCTGATGGTATTCGAACTATTGGGGTTATTACAAAGCTTGACTTAATGGACGATGGAACAGATGCTCGGGAGGTTTTAGAAAACAAACTGCTACCCCTGCGGAGAGGCTACGTGGGAGTTGTCAACAGATCTCAGAAGGACATTGAGGGGCGGAAGGACATAAAAGCTGCAATGGCAGCTGAAAGAAAATTTTTCCTGGGTCATCCAGCATACCGTCACGTTGCTGACCGCATGGGTACTCCTTACCTACAACGTGTTCTCAATCAGCAGCTCACAAACCACATCAGAGAAACCTTGCCAAGCCTTCGTGACAAACTGCAAAAGCAGCTATTAACTATGGAAAAAGAGGTTGATCAGTACAAACATTTTCGTCCTGATGATCCATCCATCAAAACTAAAGCCATGTTGCAAATGATCCAGCAGTTGCAGAATGACTTTGAGCGAGCAATTGAGGGATCAGGGTCAGCCCTAATTAGCACCAATGAGCTTTCAGGAGGAGCCAAAATTAACCGTCTCTTCCATGAGCGTTTTCCTTATGAGATTGTTCGAATGGAATTTGATGAAAAAGAACTTCGTAGAGAAATAGCTTTTGCCATTAGAAATATCCATGGCATTCGGGTTGGCTTATTTACTCCTGACATGGCATTTGATGCAATTGTTAAAGGACAAATTAGCCGACTAAAGGAACCAAGTTTAAAGTGTGTTGACCTTGTTGTGCAAGAGCTCACAAATGTAGTCCGAAACTGCTCTCTTAAGATGTCTCGGTACCCAAGACTTCAGGAAGAAACTGAAAGGATAATTACAACACACATCAGGGAACGAGAACAAATTGTTAAAGAAAATATCCTTCTAATGAATGAGTGTGAATTAGCATACATGAATACAAATCATGAAGACTTCATTGGATTTGCTAATGCCCAGCAGTCTAGTGAGAACCCCAGCAAGTCAGGTCGTAAACTGGGAAATCAAGTGATACGCAAAGGCTACATGTCAATTTCCAACCTTGGCTTTATGAAGGGTGGCTCACGTGATTACTGGTTTGTCCTAGCATCCGAGTCACTTTCTTGGTTCAAGGATGAGGACGAGAGAGATAAGAAGTACATGCTGATGCTCGATGGGTTGAAGATCAAAGATATAGAGCAAGGCTTTATGTCCCGCCGCCATGTCTTTGCCCTTTACAATCCTGATCAGAAAAATGTCTACAAGGATTACAAAGAGCTACAGTTGGGCGTTGAAACCCAAGATGACATGGACTCTTGGAAAGCATCATTCCTACGTGCTGGTGTTTACCCAGAAAAAGTTTCTGACTCTAGTAATGGAGAAGAGGGTTCTAGTGAAGGCTCCACGTCTATGGACCCTCAACTTGAACGTCAAGTGGAGACCATTCGCAACTTAGTCGATTCTTACATGAAGATTGTCACGAAGACTACACGAGATTTAGTACCAAAGATAGTCATGCATCTTATGATAAACAACACCAAAGATTTCATTATGGGGGAGCTCCTTGCCCACCTGTATGCCTCAGGTGATCAGAATTCCATGATGGAGGAGAGTCCAGAGGAGGCACGTAAACGAGAAGAAATGCTGCGTATGTACCATGCATGTAAAGAAGCACTTAAAATTATTGGAGATGTTTCCATGGCAACAGTGTCCACTCCTATGCCACCTCCAGTGAAGGATGACTGGCTCAGCACAGGCCAGGAAAGTCCAAGGATTGGTCATAGTGGGCCACCCTCTCCTGGAGGAAATATGAGACGA

Sequence No.18 *rho1*

GAATTCATGGCGGCCATACGAAAAAAATTAGTTATAGTTGGGGATGGTGCATGTGGTAAAACATGCCTTCTTATAGTATTTTCAAAAGATCAGTTCCCAGAGGTCTATGTGCCAACAGTATTTGAAAACTATGTAGCTGACATTGAAGTTGATGGTAAACAGGTAGAGTTGGCCTTGTGGGATACAGCAGGTCAAGAGGACTATGATAGACTGCGACCCCTCTCTTATCCAGACACAGATGTCATACTTATGTGCTTTTCCATTGACTCTCCAGACTCCTTAGAAAACATCCCAGAAAAATGGACTCCAGAAGTCAAACATTTTTGCCCAAATGTTCCAATTATTCTAGTAGGAAACAAAAAGGATCTACGAAATGATGCTACAACTATTAAGGAGCTTCAGAAGATGAAACAAGAGCCAGTAAAACCTGAGGAGGGACGTAACATGGCAGAGAAAATAAATGCTTTTGCTTATTTAGAGTGCTCAGCCAAGACAAAGGAGGGAGTCCGAGAAGTGTTTGAAACTGCTACCAAGGCCGCACTCTCAGTCAGGATTAAGAAGAAGACTAAATGCACCCTTTTG

**Figure S1.** The schematic diagram of the integrated rice-crayfish farming system
